# High-Intensity Focused Ultrasound Versus Airflow® in Debriding Ti-Attached *S. mutans* Biofilms

**DOI:** 10.1016/j.identj.2024.12.037

**Published:** 2025-03-22

**Authors:** Minh Dien Tran, Sheetal Maria Rajan, Hien Ngo, Amr Fawzy

**Affiliations:** Biomaterials Research Group, UWA Dental School, The University of Western Australia, Perth, Western Australia, Australia

**Keywords:** Titanium implant, Biofilms, *Streptococcus mutans*, HIFU, Ultrasound, Debridement

## Abstract

**Introduction and aims:**

Effective management of biofilm-associated peri-implantitis requires the eradication of microbial biofilms to control disease progression and preserve implant osseointegration. However, achieving complete biofilm removal remains a challenge. High-intensity focused ultrasound (HIFU) is an emerging technology offering non-ionizing radiation, no extraneous materials, and minimal residuals or aerosols. This in vitro study compared the effectiveness of HIFU and a commercial air-pressured abrasive method (Airflow®) in removing *Streptococcus mutans* biofilms attached to Titanium (Ti) discs.

**Methods:**

Bacterial biofilms were grown on 36 pairs of machined (M) and roughened (R) Ti discs for 10 days. Biofilms in the test group (12 pairs) were treated with the optimized HIFU for 2 min in a water medium and the residual biofilms were examined using two imaging methods, confocal laser scanning microscopy (CLSM), scanning electron microscopy (SEM), and three quantitative methods including crystal violet (CV), 3-[4,5-dimethylthiazol-2-yl]-2,5 diphenyl tetrazolium bromide (MTT) and flow cytometry (FCM) assays for biomass, bacterial viability, and live/dead bacterial counts respectively. The data from the test and the control samples (untreated and Airflow® treated) were subjected to ANOVA followed by post-hoc Tukey's test to determine the statistical differences between the groups.

**Results:**

Except the MTT data from M discs, the findings showed that both HIFU and Airflow® methods achieved similar levels of bacterial debridement, removing over 99% of bacteria in FCM assays (99.8 ± 0.16% versus 99.3 ± 0.49% for M discs, 99.96 ± 0.02% versus 99.5 ± 0.37% in for R discs). Complete biofilm removal was noted in HIFU-treated samples compared to the Airflow® in SEM images.

**Conclusion and clinical relevance:**

Our findings indicate that both novel HIFU and traditional Airflow® methods were equally effective in removing the *S. mutans* biofilms from titanium disc surfaces. Further research is needed to explore the clinical application of HIFU in managing peri-implantitis.

## Introduction

Peri-implantitis (PI) is defined as “a pathological condition occurring in the tissues around dental implants, characterized by inflammation in the peri-implant connective tissue and progressive loss of the supporting bone”.^1^ This condition presents a significant challenge in modern dentistry, especially as the global number of dental implants continues to grow exponentially.^2^^,^^3^

Approximately 20% of dental implants are affected by PI,^4^, ^5^, ^6^ for which the current therapeutic regimens remain suboptimal. Recurrence is common and often results in implant failure.^7^^,^^8^ Microbial biofilms attached to implant surfaces are widely recognized as a primary cause of PI,^9^ making disrupting or removing of these complex microbial structures the cornerstone of effective management.

Owing to the intrinsic microbial resistance of multispecies oral biofilm communities,^7^ physical biofilm debridement methods have been extensively studied. Although an overview of systemic reviews by Dos Santos Martins et al,^10^ highlighted the superiority of surgical approaches compared to non-surgical protocols, implant surface decontamination/debridement continues to be a fundamental aspect of disease management.^10^^,^^11^ Nonetheless, this fundamental therapeutic approach has encountered numerous challenges.^8^^,^^12^ Notably, the authors described how hand tools (titanium, plastic, Teflon, and carbon fiber) and mechanical instruments (abrasive air power systems, rubber cups, and sonic tips) were effective as mainstream nonsurgical approaches to reduce the clinical signs of PI, but these could not eliminate the disease. The use of air power abrasives (sodium bicarbonate, sodium hydrocarbonate, or the amino acid glycine) such as Airflow®, has been shown in *in vitro* and *in vivo* studies to effectively remove the bacterial endotoxin and biofilm^13^; however, concerns have been raised regarding aerosol generation, residual powder remaining on the implant surface, and the potential for subcutaneous emphysema and epithelial desquamation.^13^ Similarly, the use of metal ultrasonic scalers with metal tips could reduce the dental plaque score and create a smoother implant surface; however, the plaque reduction was found to be modest (73% to 53%) and hyperthermic side effects on dental implants were observed.^13^

In addition to such treatments for managing PI, other treatment methods, including the topical application of chlorhexidine, lasers, photodynamic therapy, and systemic probiotics, have been considered adjuncts and require further investigation.^8^^,^^12^^,^^13^ Nevertheless, the plethora of current treatment methods for PI suggests that a variety of clinical strategies is necessary to manage the condition. To address this objective, high-intensity focused ultrasound (HIFU) presents a novel approach for the debridement of dental implants in managing PI. This technology offers benefits over existing methods in that it is devoid of radiation and aerosols, requires no additional materials, and can effectively reach inaccessible irregularities of the target.

Since the first article published in 1927 by Wood and Loomis,^14^ HIFU has been successfully implemented to treat a variety of medical conditions, including prostate cancer, Parkinson's disease, and neuropathic pain^15^ by exploiting its mechanical ablation and thermal coagulation properties.^16^^,^^17^

Although HIFU is widely used in medicine, its application in dental research and clinical therapy remains underdeveloped, despite a growing interest in this field.^18^ While microbiology studies have demonstrated that HIFU can inhibit *S. mutans* growth on dentin^19^ and effectively eliminate or disrupt *E. coli* and *E. faecalis* biofilms,^20^^,^ ^21^ its potential application in detaching bacteria from dental implants remains inadequately explored. To address this scientific gap, this study aimed to investigate the effects of HIFU on the removal of bacterial biofilms attached to Ti substrates. Building on this scientific foundation, we hypothesize that HIFU is equally effective as Airflow® in removing *S. mutans* biofilms from titanium surfaces.

## Materials and methods

### Study design

#### Ti substrates

36 pairs of Ti discs (10 mm in diameter, 78.54 mm^2^, 2 mm in thickness) produced by Southern Implants (Pty Ltd., Irene, South Africa) were used to study the removal/debridement effects on *S. mutans* attached biofilms in this study. The testing side of the discs, where biofilm growth took place, was machined (M) on 36 discs and roughened (R) using alumina grit blasting on 36 discs. The remaining non-testing area of the disc was polished to minimize bacterial adhesion.

In addition, one pair of Ti discs and one pair of Ti implants (4mm in diameter, 9mm in length) by the same manufacturer [DCT4009 DC Unroughened (machined – M) and DCT4009 DC (roughened – R)] were used for surface characterization, and four R discs for HIFU optimization bring the total substrates to 80 including 78 Ti discs (37 M and 41 R) and two (one M and one R) Ti dental implants.

#### Study design

**Fig. 1:**
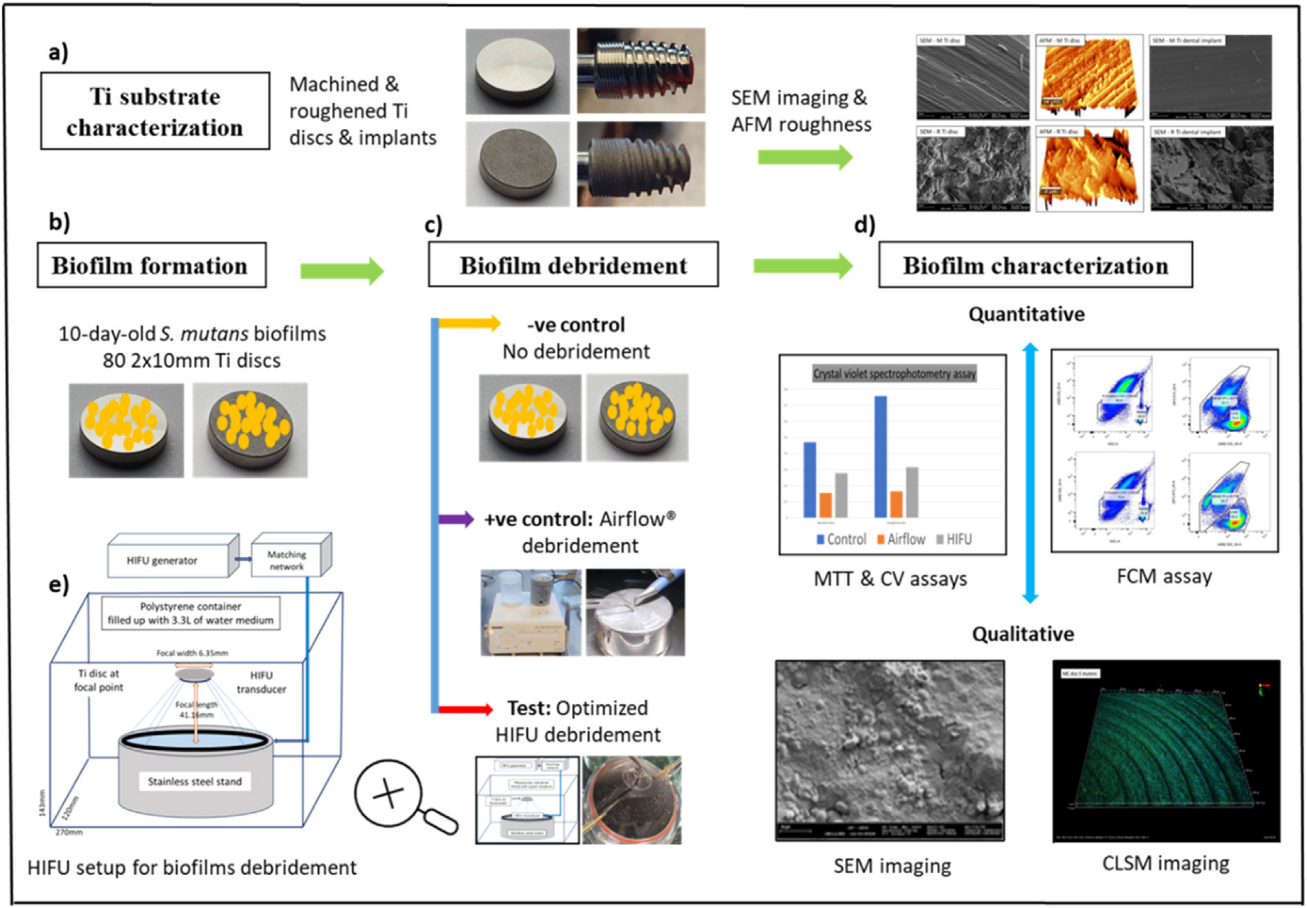
Study design illustrating (a) characterization of Ti disc microscopic roughness using AFM and comparing their SEM microscopic topography with Ti implants from the same manufacturer. (b) *S. mutans* biofilm culture. (c) biofilm debridement process and (d) characterisation of residual biofilms using both qualitative and quantitative methods. Details of HIFU set up also provided (e).

The study design is graphically summarized in Figure 1 showing the four interconnected investigation stages: firstly, surface characterization of Ti discs represented by three atomic force microscopy (AFM) roughness parameters Sa, Sq and, Sdr. The disc microscopic topography was also examined in this step using scanning electron microscopy (SEM) and their images were compared to those of commercial Ti implants from the same manufacturer; Secondly the culture of *S. mutans* biofilms; Thirdly the debridement experiment protocol; And fourthly the characterization methods of residual biofilms after treatment. Figure 1 also describes the details of the HIFU setup.

To answer the research question, two qualitative imaging (CLSM and SEM) and three quantitative methods (MTT, CV, and FCM) were employed to characterize the biofilms. While CLSM and SEM images were aimed at displaying the topography of the biofilms in relation to Ti surfaces the quantitative; MTT, CV and FCM assays were purposed to reveal respectively the metabolic activity, biomass, and number of bacteria that remained attached to Ti surfaces after treatments. The results were compared with the negative controls where the biofilms were left untreated and positive controls in which the biofilms were removed using the current clinical method of air-abrasive debridement named Airflow® (Figure 1).

### HIFU setup

A HIFU transducer (H-115, S/N: 046, Sonic Concept, USA), with 64.00 mm in diameter, 41.16 mm focal length and 6.35 mm focal width, was connected to a TPO-200-09 HIFU generator (Sonic Concept, USA – 33 W maximum intensity) through a XDR094-014 fundamental RF impedance matching network at 250 kHz frequency with 2 min active followed by 2 min idle in continuous mode. The transducer was placed on a stainless-steel stand in a rectangular polystyrene container (270×143×120 mm dimension) filled with 3.3 L of distilled water which fully submerged the transducer to 10 mm above the focal point (Figure 1). To apply HIFU, the disc was hoovered slowly over the focal area for 2 min with the biofilms facing the incoming HIFU beams, while the generator was in active mode. To select the optimal HIFU intensity, HIFU at 20 W and 30 W was applied to two pairs of R discs with attached biofilms. The residual biofilms were imaged using CLSM and quantified using FCM, and 30 W was selected because of its higher debridement effects.

According to the equation *MI = P_ra_ /* $\sqrt{fc}$^22^ where MI represents the mechanical index, *P_ra_* denotes the peak negative pressure focus at 4.95 MPa, and *fc* is the central frequency of 0.250 MHz, the calculated maximum MI value is 9.9, which is much higher than the diagnostic mechanical index of 1.9.^22^

### Characterization of Ti surface

To quantitatively characterize the Ti surface, the roughness and topography of a pair of (1M + 1R) Ti discs were analysed using Atomic Force Microscopy (AFM) (WITec alpha 300RA+ - Germany). The AFM was performed at high resolution in the tapping mode using a silicon cantilever coated with aluminium. Areas of 20 µm x 20 µm were randomly selected and scanned with a resolution of 512 lines and speed of 2 s. Data were collected and analysed using Project5 5.2 (WITec Suite FIVE Software, Germany). The background subtraction Sa (arithmetic mean height), Sq (root mean square height), and Sdr (developed interfacial area ratio) nanoscopic roughness parameters from the eight disruption–free scans were extracted from the topography mode for data analysis.

To qualitatively characterize the testing surface, a pair of M and R discs and two M and R implants were affixed onto aluminium SEM stubs using Cu tape, carbon-coated (∼20 nm) (Polaron SC7640, Quorum Technologies Ltd, U.K), and examined using Scanning Electron Microscopy with a Zeiss 1555 VP-FE SEM. Ten sites were randomly selected for the examination of each sample. SEM images were captured at 5000x magnifications, and the images obtained from the discs were compared with implants of similar surface morphology, that is, M to M and R to R.

### Biofilm formation on Ti discs

*S. mutans -* from The American Type Culture Collection (ATCC) 700610 - were grown overnight in Brain-Heart Infusion broth (BHI) (Sigma-Aldrich, Australia) supplemented with 1% sucrose (Sigma-Aldrich, Australia) at 37°C for 24 h and adjusted to a concentration of 5×10^6^ colony-forming units (CFU) per mL (optical density at 600 nm = 0.5) (McFarland Densitometer – Fisher Biotec Australia).

To grow the biofilms [modified protocol from Lemos et al^23^ and ATCC bacteriology culture guide], the Ti discs were placed individually in two 24-well plate filled with 2 mL of adjusted bacterial suspension and incubated at 37°C non-shakingly for 2 days followed by 8 days in an orbital shaker at 50 rpm, the medium was replenished every two days. On the 10^th^ day, Ti discs were washed gently in 2 mL of phosphate-buffered saline (PBS) to remove loose bacteria and kept in 2 mL of PBS until treatment.

For each experiment, biofilms were cultured on 12 pairs of Ti discs (12 M + 12R) in two 24-well plates, with six pairs each. One group (six pairs) was dedicated to FCM, whereas the other (six pairs) was used for the remaining four analytical methods: MTT, CV, CLSM, and SEM (Figure 1). Each group was further divided into negative control, positive control, and test subgroups of two pairs (2M + 2R). No treatment was applied to negative controls (C) while biofilms in the positive controls (A) were removed for two minutes using Airflow® (EMS Air-Flow Master Piezon® - Swiss Vallée de Joux, Switzerland), which delivered a grit blasting jet mix of water, air, and Erythritol Amorphous Silica 14 µm particles (Airflow plus Powder) following the manufacturer's instructions. The same application time (2 min) was used in the test (HIFU) groups at 30 W. The treated discs were gently dipped four times in 2 mL of PBS to remove debris and placed in 2 mL of fresh PBS for analysis. The experiment was then repeated twice, resulting in a total sample of 36 pairs.

### Characterization of biofilms debridement

#### Quantitative characterization

Three methods of CV, MTT, and FCM were used to measure the three aspects of bacterial biofilms including their biomass, metabolic activity, and number of embedded “live” bacteria respectively.

To release the biofilms from the attached Ti surfaces, the samples were placed in 5 mL flat-bottom tubes filled with 2 mL of PBS and placed in an ultrasonic bath (L&R SweepZone Technology, NJ, USA) for 30 min. The tubes were then shaken using a vortex mixer for 2 s, the 2 mL suspension was extracted. In the FCM group, the entire 2 mL volume was used for analysis and in the other group 1 mL each for the MTT and CV assays.

For bacterial counts using FCM**,** the 2 mL suspension was centrifuged at 4000 rpm for 10 min, the supernatant was removed and 2 mL of sodium chloride 0.9% was added to the tube. After vortex mixing, 100 µL of this bacterial suspension was extracted for staining with SYTO9 and propidium iodide from LIVE/DEAD™ BacLight™ Bacterial Viability and Counting Kit (Thermo Fisher Scientific) following manufacturer's instructions (1.5µL propidium iodide +1.5µL SYTO9 + 10µL microspheres) producing 1,000 µL of stained bacterial suspension for measurement. Four single-color controls were prepared and the results from these controls were used for voltage calibration and gating. An LSR Fortessa cytometer and BD FACSDiva^TM^ software (BD Biosciences) were used with laser wavelength of 488 nm and 561 nm, respectively. The fluorescence signals were collected in three channels with filters at 610 nm (for the 561 nm laser), 530 nm, and 695 nm (for the 488 mm laser). Forward scatter, side scatter and fluorescence data were collected for 5 min using a medium flow (250µL in total) with logarithmic signal amplification and the files were saved in FCS (flow cytometry standard) format and analysed using FlowJo v10 (BD Biosciences) software. The sample quality was controlled using the inspection function of the software, the FSC versus time plot, and the number of microspheres (10^6^ in each 1mL sample). Four single-color controls were used for gating, which differentiated three populations SYTO9 stained (live), PI-stained (dead), and total bacteria. The numerical counts of the +veSYTO9 population were extracted and the percentage of the population reduction by Airflow® and HIFU treatments compared to negative controls was calculated and used for statistical analysis.

To quantify the biofilm biomass, samples for CV assays were prepared according to O'Toole^24^ and the absorbance at 550 nm recorded using microtiter plate reader (Sunrise™, Tecan, Switzerland) and Magellan™ 7.2 software (Tecan, Hombrechtikon, Switzerland). To further investigate the metabolic activity of the viable bacteria, MTT assays were performed with Cell Proliferation Kit I (MTT) (Roche/Sigma-Eldrich – Mannheim, Germany) according to the manufacturer's instructions and Fernandes et al^25^ The absorbance at 570 nm was collected using the same software and reader.

#### Qualitative characterization

Two imaging methods (CLSM and SEM) were used to examine the “live/dead” embedded bacteria, the nature of the biofilms and the cleaning pattern on the positive and test samples.

For CLSM, the remaining biofilm on each Ti disc was stained with LIVE/DEAD™ BacLight™ following the manufacturer's instructions - 1.5 µL of propidium iodide and 1.5 µL of SYTO9 in 2 mL of PBS for 15 min. The samples were then gently dipped four times in 2 mL of PBS to remove excess stain, fixed in 2 mL of 4% formaldehyde solution for 30 min, and washed with PBS. The stained biofilms were mounted on glass cover slip (0.17 mm thick, reflective index of 1.0) and images were acquired using a Nikon A1RMP confocal and multiphoton microscope. Five sites were selected from each sample. Images of the Ti surfaces were captured first using the reflection mode, followed by photographing the attached biofilm using the fluorescent method. In reflection mode, Plan Apo VC 20x objective lens, 405.0 nm excitation emitting 575.0 nm wavelengths, 26.82 Airy Unit pinhole, 0.21µm pixel size, and 1024×1024-pixel scan area parameters were used, while 488.0 nm and 561.0 nm laser excitation emitting 525.0 nm green and 595.0 nm red wavelengths respectively, were used in the fluorescent mode. The images were processed using NIS-Element AR software by projecting full-view snapshots (8-bit GRB) of all channels, which were then copied into Microsoft® PowerPoint® (Version 2405) for presentation without any modification in colour, sharpness, or contrast.

For SEM, the samples were platinum-coated (Polaron SC7640; Quorum Technologies Ltd., U.K) after CLSM acquisition. Fives sites were selected for examination from each disc, and the SEM images were captured at 5000x magnification using 5 kV voltage, 30 µm aperture, and secondary electron detector from a Zeiss 1555 VP-FESEM (high-resolution, field-emission variable-pressure scanning electron) microscope.

### Statistical analysis

Data from these three quantitative measurements were processed using Microsoft Excel software version 2410 (Build 18129.20116). All data are presented as the mean ± standard deviation. ANOVA followed by post-hoc Tukey's test was performed to determine the statistical differences among the groups at the alpha level (*P* < .05).

## Results

### Ti surface characterisation

**Fig. 2:**
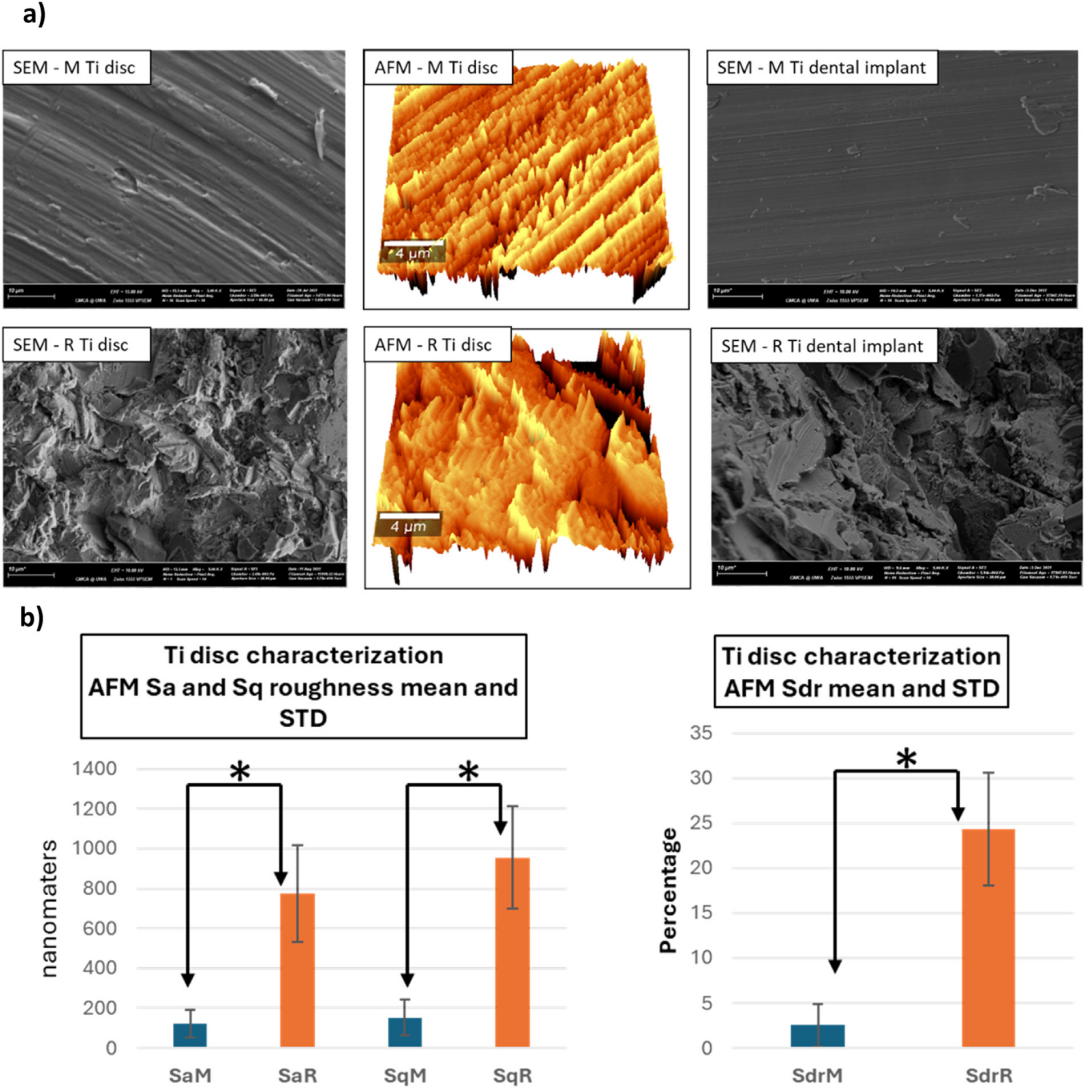
(a), x5000 magnification SEM images and 3D AFM projections of M and R surfaces. The M implant showed less irregularities and was cleaner compared to M disc. The R surfaces of both implant and disc had similar topography and displayed high levels of roughness. (b), AFM Sa and Sq roughness from R discs were 6.4 and 6.2 times higher than these parameter from M discs, respectively, and Sdr was 9.7 times higher in R discs – a statistically significant difference *.

SEM images of M disc (Figure 2A) were characterized by the concentric cutting lines of 1 micron apart with noticeable level of heterogenicity in depths, width, and continuity. Debris, scratch lines, and voids were also observed. SEM images of R discs exhibited high levels of irregularity with sharp troughs and peaks and the debris was less distinguishable on rough backgrounds. Although the R disc and the implant shared topographical similarities, the M implant surface appeared smoother and cleaner than that of the M disc (Figure 2A). AFM measurements showed high statistical significance difference between the two surfaces that the roughened Ti substrate had average Sa and Sq of 774 nm and 954 nm, respectively, which were more than six times higher than those of the M surface. The Sdr of the R surface was 24%, which is almost 10 times higher than that of M (Figure 2B).

### Biofilm characterization

At the end of data collection, biofilms from 36 pairs of Ti discs were examined, including 18 pairs for the FCM assays, nine pairs for MTT/CV assays, and nine pairs for CLSM/SEM imaging.

#### Quantitative biofilm characterization

**Fig. 3:**
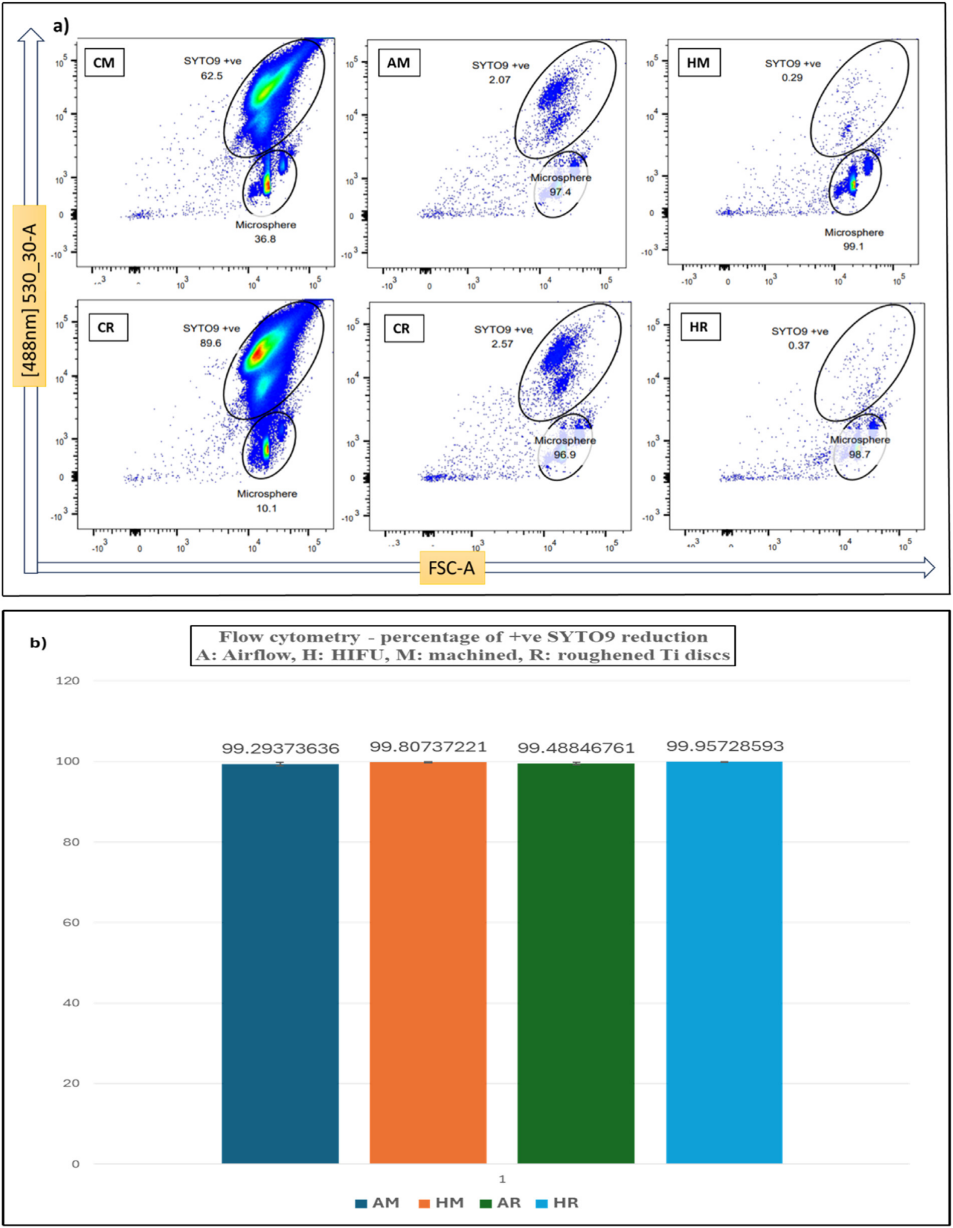
(a), Pseudo colour plot showing the +ve SYTO9 bacterial populations of three sample groups - -ve control (C), Airflow® (A) and HIFU (H) treated - on the forward scatter area (FSC-A) versus green colour channel (488 nm) axes. (b), The percentage of reduction in the population by Airflow and HIFU treatments compared to the -ve controls exceeded 99% and no statistical difference between samples treated with Airflow® and those with HIFU.

**Fig. 4:**
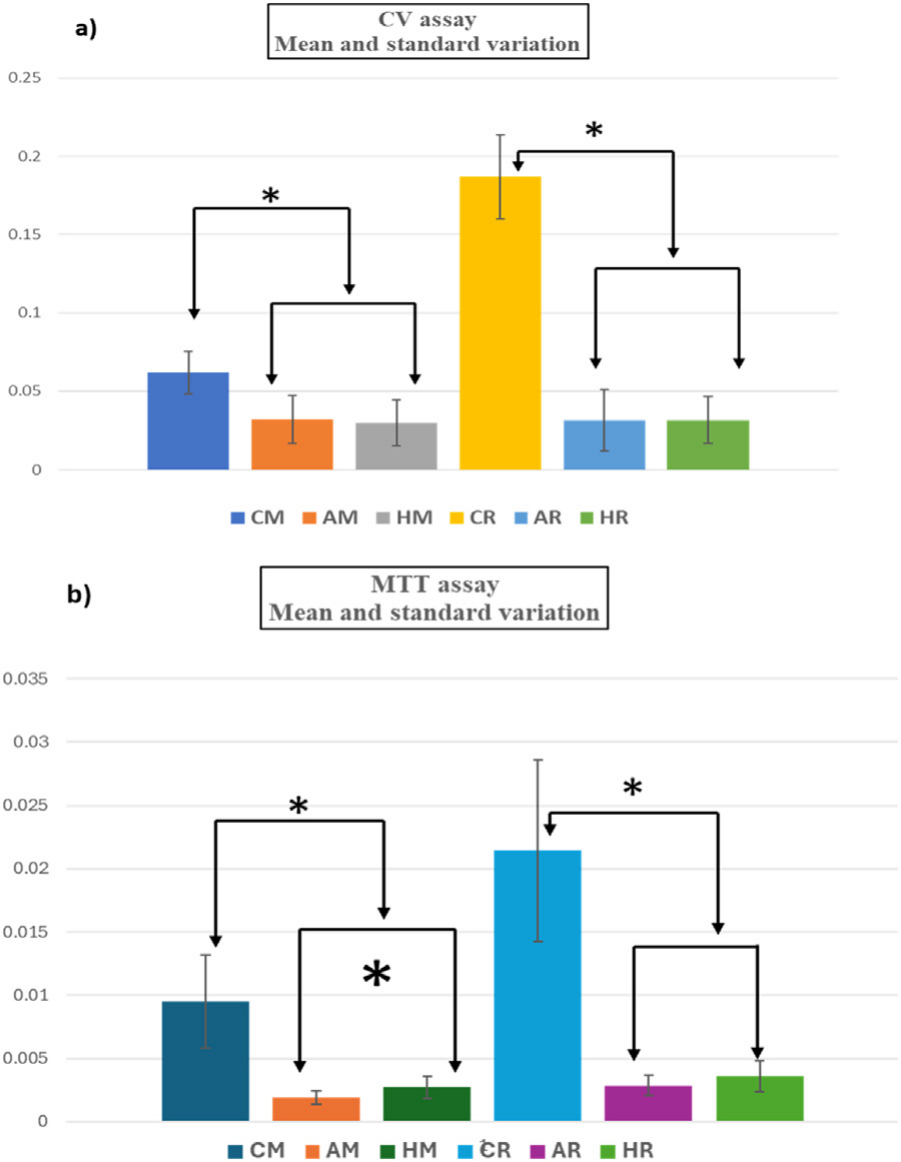
CV (a), and MTT assays (b), of residual *S. mutans* biofilms after debridement with Airflow (A) and HIFU (H). Although there was statistically higher reading from -ve controls compared to +ve and test samples, the statistically significant difference between samples treated with Airflow® and HIFU was ONLY observed in the MTT assay and from M discs (*).

Figure 3 demonstrates the results from FCM assays while Figure 4 represents those from CV and MTT assays. The gated pseudo colour plots of forward scatter area versus 488nm channel axes (Figure 3A) show statistically significant higher +ve SYTO9 counts from biofilms grown on R discs (451,581 on average) compared to those from M discs (205, 648 on average). After debridement with either HIFU or Airflow®, more than 99% of the +ve SYTO9 bacterial population was removed compared to that of -ve controls (Figure 3B). and there was no statistical difference detected between these two methods of biofilm removal.

Figure 4 shows consistently higher reading from the untreated (C) samples compared to treated either with Airflow® or HIFU in both MTT and CV assays from both M and R surfaces. However, there was no statistical difference between the treated groups except in MTT assay from M surfaces in which the HIFU treated samples showed higher MTT reading.

#### Qualitative biofilm characterization

**Fig. 5:**
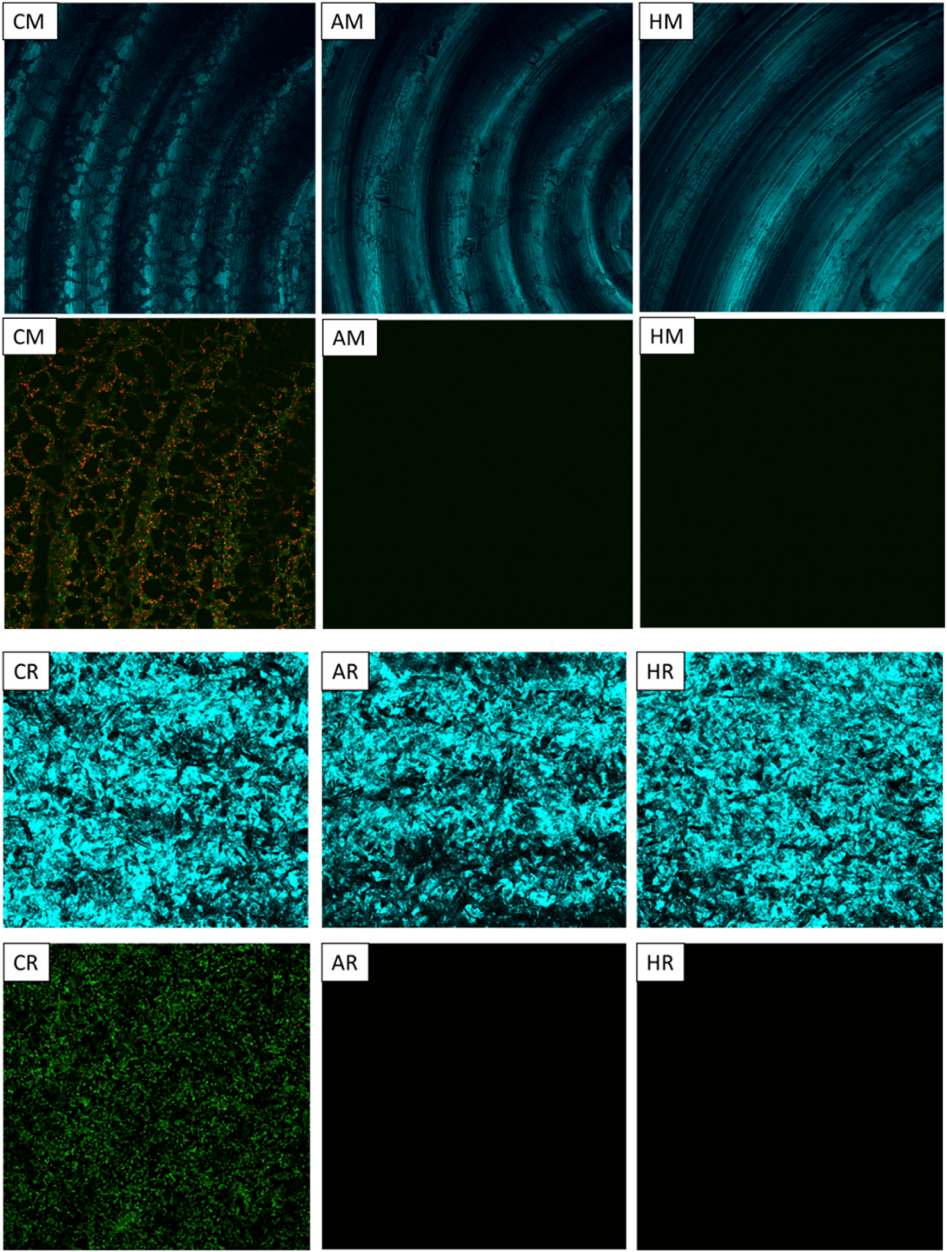
Fluorescent CLSM images (second and fourth rows) showed the denser and greener negative control biofilms on roughened (R) surfaces almost zero fluorescent signals emitting from samples treated with either Airflow® (A) or HIFU (H). This poor emission severely hindered the sample focusing procedure. Therefore, to locate the Ti attaching surfaces, reflection CLSM techniques were used (as shown in first and third rows).

**Fig. 6:**
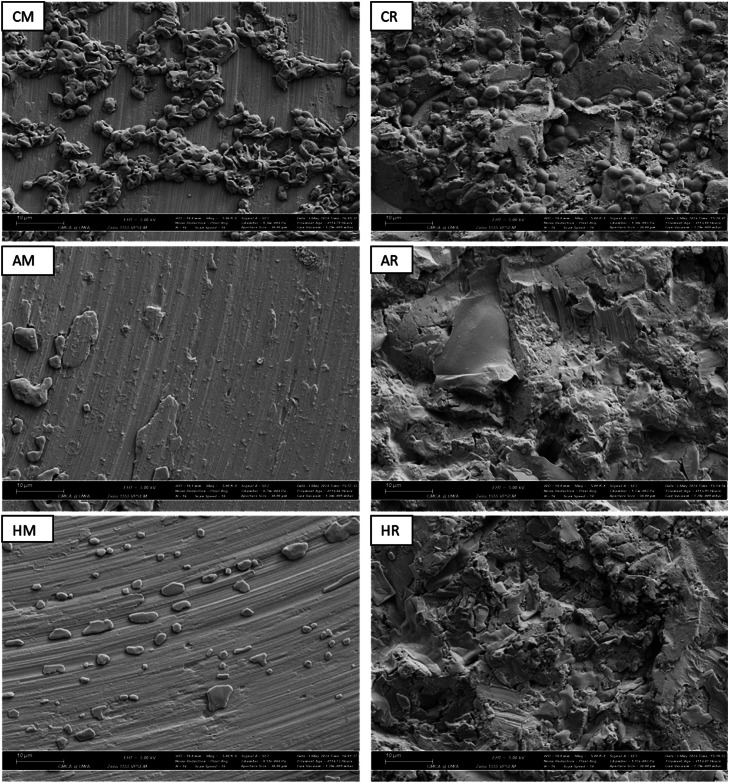
x5000 magnification SEM images displayed the abundance of healthier and better-connected *S. mutans* bacteria on untreated roughened (CR) compared to machined surfaces (CM). However, the biofilms did not cover the entire substrate in both surface morphologies. Although bacteria were undistinguishable on treated samples, fragments of biofilms remained. In HIFU samples (HM), there were areas of total biofilm detachment exposing microscopic topography of the surfaces while Airflow® treatment (AM) left a thin layer of mesh-like material obscuring their microscopic characteristics.

CLSM and SEM images of the biofilms are presented in figures 5 and 6. Fluorescent CLSM images of treated samples with either Airflow® or HIFU showed almost zero light emissions. This fluorescent darkness made the locating and focusing process onto biofilms highly challenging, hence, the reflection technique was applied and able to identify the substrates surfaces and even display the biofilms on M surfaces (Figure 5). In SEM, the R surfaces, with their higher roughness, irregularities, and area, inhabited larger attached bacteria that appeared more encapsulated, interconnected with each other into clusters, and showed more mitotic images. It was also interesting to point out the surface cleanliness patterns observed on SEM in which HIFU seemed to remove biofilms by an ablating mechanism, leaving areas completely devoid of biofilms, while Airflow appeared to work by wiping action, leaving a thin layer of biofilm substances painted over the Ti surfaces (Figure 6).

In summary, the results showed quantitatively and qualitatively that HIFU had comparable effects to Airflow® - a clinical air-abrasive system - in detaching 10-day-old *S. mutans* biofilms grown on machined and roughened flat Ti surfaces which shared similarities with commercial Southern Implants Ti dental implants. Although the results validated the fact that bacteria were not removed completely regardless of the technique, the surfaces cleaned by HIFU displayed areas devoid of total biofilm.

## Discussion

Although the impact of HIFU on hard dental tissues has been demonstrated,^19^, ^20^, ^21^^,^^26^, ^27^, ^28^, ^29^, ^30^, ^31^ its effects on biofilms attached to titanium surfaces have not been explored. This study aimed to fill this gap and pave the way for further research on HIFU applications to remove and destroy biofilms on Ti surfaces, which are pathogenic in peri-implantitis,^1^^,^^4^, ^5^, ^6^^,^^9^^,^^32^, ^33^, ^34^, ^35^ a condition for which no ideal non-invasive therapy currently exists.^7^^,^^8^^,^^10^ To achieve this goal, the primary objective was to investigate the debridement effect of HIFU on *S. mutans* biofilms attached to flat Ti surfaces that mimic the surface topography of Ti dental implants. The findings, gathered through multiple qualitative and quantitative measurements accept the hypothesis that HIFU (in this study setting) is as effective as the current clinical method of Airflow® (an air-powered abrasive technology) - in detaching *S. mutans* biofilms adhered to both machined and alumina blasted roughened Ti surfaces.

The equal debridement capability of HIFU compared to Airflow® in this study can be explained by the higher calculated maximum negative pressure of 9.9 Mpa, compared to other studies using 4.1 to 7.6 Mpa value,^20^ leading to high ablation effects. Another factor to consider is the expansive focal area (31.67 mm^2^) of the transducer, coupled with sturdy landmarks that assist in accurately positioning the targeted biofilm (78.54 mm^2^ area) at the focal point. At this high intensity, hyperthermic effects are a concern; however, our internal investigations found that the temperature increase did not exceed 5ᵒC in this high-volume water medium setting.

Despite the lack of direct evidence^33^ associating *S. mutans* to PI, this bacterium is present in subgingival biofilms collected from patients with healthy gingival conditions and periodontal disease.^36^ These streptococci are facultative anaerobes with a fast exponential growth rate of 0.24 h^−1^^37^ and have been found to be important pioneer colonisers during biofilm formation.^38^

Airflow® was selected as the positive control in this investigation due to its established use in clinical applications for debriding implant biofilms both supra- and subgingivally. Although several scientific studies and reviews have documented its efficacy,^39^, ^40^, ^41^, ^42^, ^43^ our study combined multiple imaging and quantitative measurement methods, including FCM and CLSM reflection techniques. Considering the complex and dynamic nature of bacterial biofilms and the lack of universal characterization methods and training,^44^ this combination is essential.

The dual fluorescence and reflection CLSM technique was first used by Baschong et al in 2001^45^ to image Ti implant-tissue interfaces. This technique was revised,incorporated into our research protocols and proven particularly valuable for visualizing adherent biofilms and their distribution in relation to Ti surface topography.

Given the novelty of HIFU, there is currently a lack of comparable studies in the literature. However, related research by Vyas et al^47^^,^^48^ which utilized cavitation generated by oscillating ultrasonic scaler tips, and an investigation by Yamada et al,^49^ which applied cavitation jets, demonstrated the potential of these methods to remove bacterial biofilms. Nonetheless, these studies did not employ a comprehensive approach combining multiple quantitative and qualitative analyses.

The surface (being a crucial element in the entire structure of Ti dental implants) plays a pivotal role in molecular interactions and cellular responses leading to osseointegration.^46^, ^47^, ^48^ Various surface roughening and modification methods have been proven to accelerate the process^46^, ^47^, ^48^; An intriguing study by Lima et al^49^ investigated the affinity of fibroblasts and pre-osteoblastic cells for different Ti surfaces, including machined, acid-etched, and anodized surfaces, shedding light on complex interactions between cells and adhering substrates. The anodized group demonstrated the highest cell viability for Balb/c3T3 fibroblasts, while the acid-etched group exhibited the highest cell viability for MC3T3-E1 lineage pre-osteoblasts. In the context of peri-implantitis, there are conflicting results regarding the role of implant surface roughness in the risk of peri-implant bone loss and PI.^50^^,^ ^51^ A systematic review by Saulacic et al^51^ indicated that machined implants were associated with higher plaque scores and peri-implant bone loss than rough-surface implants, while Jordana et al^50^ showed convincing evidence of the association between the level of implant roughness and the risk of peri-implantitis in their systematic review and meta-analysis. In our study, the roughened titanium surfaces clearly supported healthier and more abundantly embedded *S. mutans*. However, further research is necessary to evaluate the growth of various PI-related bacterial biofilms on different titanium surfaces.

Teixeira et al^52^ demonstrated the inability of metronidazole gel to prevent bacterial and fungal infiltration (*Aggregatibacter actinomycetemcomitans, Prevotella melaninogenica, Bacteroides fragilis*, and *Candida tropicalis*) through the Morse taper implant-abutment interface. In their discussion, the authors also highlighted the failure of other antimicrobial agents and mechanical seals to achieve the same goal, attributing this to the significant intrinsic gap (ranging from 2.3 to 100 µm) between implants and abutments. The findings from this study reinforce the importance of mechanical biofilm debridement in the management of peri-implantitis. The principle of debridement was also emphasized in the context of bone tissue regeneration in peri-implantitis, as demonstrated by a systematic review of randomized clinical trials conducted by Castro et al^12^. Given the characteristics of HIFU, the technology, once refined, could be utilized during the decontamination phase preceding regenerative procedures in the surgical management of PI.

The SEM results reveal the unexpected distinct differences in debriding patterns between HIFU and Airflow®. HIFU shows the ability to ablate and create areas clear of debris, whereas Airflow® appears to "wipe over” the biofilms leaving behind a mist of residuals to which concerns have been raised.^13^ Although additional research is needed to establish the clinical significance of this phenomenon, complete biofilm removal is preferred to reveal uncontaminated Ti surfaces, which are optimal for promoting tissue adherence and regeneration. Given the unique working mechanism of HIFU, where sound waves can energize the entire three-dimensional transmission medium at the microscopic level, it is predictable that this form of ultrasound would impact the morphologically complex surface of dental implants.

Results from this study indicate that HIFU has the potential to become a non-invasive therapeutic modality for the management of peri-implantitis. In addition, our findings also contribute to the existing literature by highlighting the potential roles of dual fluorescence-reflection CLSM and FCM in the study of dental implant-attached biofilms.

The limitations of this study include the *in vitro* nature, the limited number of substrates available, and the flat surfaces used for experimentation. Dental implants, with their complex macro- and microscopic surface morphology, promote the colonization of microorganisms and significantly complicate the debridement process.

Immediate research should involve conducting experiments on multi-species peri-implantitis pathogenic bacteria,^33^ particularly anaerobic strains, such *as Porphyromonas gingivalis* and *Fusobacterium nucleatum*, within the context of peri-implant pocket models. These efforts aim to advance HIFU research toward animal studies and human trials.

In relation to bone effects, HIFU has been utilized for ablating bone tumours and managing pain caused by bone lesions.^53^ However, its impact on osseointegration remains unexplored, unlike other emerging technologies such as photobiomodulation. This technique has been systematically reviewed and meta-analysed by Saini et al,^54^ demonstrating the potential of low-level laser therapy to enhance implant osseointegration, as measured by Periotest. A recent publication on the acceleration of dentine remineralization using HIFU^55^ has further expanded the understanding of HIFU's applications in dentistry,^18^ highlighting its prospective as a versatile dental multitool.

Regarding biofilm debridement, this *in vitro* concept-proving study qualitatively and quantitatively showed that HIFU was effective in removing *S. mutans* grown on Ti discs, whose surface composition and topography were comparable to those of commercial Ti implants. However, considerable number of further studies are required to convert HIFU into a clinically applicable device from bioengineering perspectives, including device configuration, transmission media, HIFU parameters, debridement effectiveness, and adverse effects, particularly hyperthermia. A handpiece-like HIFU delivery system incorporating a HIFU transducer, immersed in water as both a transmission medium and coolant, has been envisioned as a potential clinically applicable tool and proposed for development in the next phase of research.

## Conclusion

Within the constraints of this study, the results confirmed the hypothesis that HIFU was equally effective as Airflow® in removing *S. mutans* biofilms from both machined and roughened Ti surfaces. With its demonstrated benefits, this technology merits further exploration and shows potential as a non-invasive solution and a complementary tool in surgical therapies for the management of PI, a rising concern in contemporary dentistry.

## Conflict of interest

None disclosed.
